# *CHRNA2* and Nocturnal Frontal Lobe Epilepsy: Identification and Characterization of a Novel Loss of Function Mutation

**DOI:** 10.3389/fnmol.2019.00017

**Published:** 2019-02-12

**Authors:** Chiara Villa, Giulia Colombo, Simone Meneghini, Cecilia Gotti, Milena Moretti, Luigi Ferini-Strambi, Elisa Chisci, Roberto Giovannoni, Andrea Becchetti, Romina Combi

**Affiliations:** ^1^School of Medicine and Surgery, University of Milano – Bicocca, Monza, Italy; ^2^Department of Biotechnology and Biosciences, University of Milano – Bicocca, Milan, Italy; ^3^CNR, Institute of Neuroscience, Milan, Italy; ^4^Department of Medical Biotechnology and Translational Medicine, University of Milan, Milan, Italy; ^5^Department of Clinical Neurosciences, San Raffaele Scientific Institute, Sleep Disorders Center, Vita-Salute San Raffaele University, Milan, Italy

**Keywords:** ADNFLE, ADSHE, genetics, frontal lobe epilepsy, nicotinic receptor, patch-clamp

## Abstract

Mutations in genes coding for subunits of the neuronal nicotinic acetylcholine receptor (nAChR) have been involved in familial sleep-related hypermotor epilepsy (also named autosomal dominant nocturnal frontal lobe epilepsy, ADNFLE). Most of these mutations reside in *CHRNA4* and *CHRNB2* genes, coding for the α4 and β2 nAChR subunits, respectively. Two mutations with contrasting functional effects were also identified in the *CHRNA2* gene coding for the α2 subunit. Here, we report the third mutation in the *CHRNA2*, found in a patient showing ADNFLE. The patient was examined by scalp EEG, contrast-enhanced brain magnetic resonance imaging (MRI), and nocturnal video-polysomnographic recording. All exons and the exon-intron boundaries of *CHRNA2*, *CHRNA4*, *CHRNB2*, *CRH*, *KCNT1* were amplified and Sanger sequenced. In the proband, we found a c.754T>C (p.Tyr252His) missense mutation located in the N-terminal ligand-binding domain and inherited from the mother. Functional studies were performed by transient co-expression of α2 and α2^*Tyr*252*His*^, with either β2 or β4, in human embryonic kidney (HEK293) cells. Equimolar amounts of subunits expression were obtained by using F2A-based multi-cistronic constructs encoding for the genes relative to the nAChR subunits of interest and for the enhanced green fluorescent protein. The mutation reduced the maximal currents by approximately 80% in response to saturating concentrations of nicotine in homo- and heterozygous form, in both the α2β4 and α2β2 nAChR subtypes. The effect was accompanied by a strong right-shift of the concentration-response to nicotine. Similar effects were observed using ACh. Negligible effects were produced by α2^Tyr252His^ on the current reversal potential. Moreover, binding of (±)-[^3^H]Epibatidine revealed an approximately 10-fold decrease of both K_d_ and B_max_ (bound ligand in saturating conditions), in cells expressing α2^Tyr252His^. The reduced B_max_ and whole-cell currents were not caused by a decrease in mutant receptor expression, as minor effects were produced by α2^Tyr252His^ on the level of transcripts and the membrane expression of α2β4 nAChR. Overall, these results suggest that α2^Tyr252His^ strongly reduced the number of channels bound to the agonist, without significantly altering the overall channel expression. We conclude that mutations in *CHRNA2* are more commonly linked to ADNFLE than previously thought, and may cause a loss-of-function phenotype.

## Introduction

ADNFLE, also known as autosomal dominant sleep-related hypermotor epilepsy (ADSHE) (Tinuper et al., 2016) is a familial idiopathic focal epilepsy with increased nocturnal instability (Sansoni et al., 2013), characterized by a wide spectrum of brief stereotyped hypermotor seizures, mostly occurring during non-rapid eye movement (non-REM) sleep. About the 80% of individuals develop ADNFLE in the first two decades of life and mean age of onset is 10 years (Nobili et al., 2014; Tinuper et al., 2016). Within a family, the manifestation of the disorder may vary considerably, and no clear difference between sexes is observed.

ADNFLE was the first epilepsy to be recognized as a channelopathy, i.e., a disease resulting from ion channel dysfunction, after the identification of the first mutation in the *CHRNA4* gene, coding for the α4 nAChR subunit (Steinlein et al., 1995). Subsequently, evidence has grown about the role of nAChRs in the pathophysiology of ADNFLE (Ferini-Strambi et al., 2012). Nonetheless, mutations in nAChR genes are rare and the involvement of other genes implicated in ADNFLE has been recognized since 2005 (Combi et al., 2005b). In fact, mutations were also found in *KCNT1* (coding for a sodium-dependent K^+^ channel) (Heron et al., 2012) as well as in genes not coding for ion channels, such as *CRH* (corticotropin-releasing hormone) (Combi et al., 2005a) and *DEPDC5* (Disheveled, Egl-10 and Pleckstrin Domain-containing protein 5) (Ishida et al., 2013).

The nAChR is a pentameric ion channel formed by various combinations of α and β subunits, which determine the physiological and pharmacological properties of each subtype (Dani and Bertrand, 2007). Most ADNFLE mutations of the nAChR were found in the genes coding the α4 (Steinlein et al., 1995), and β2 (De Fusco et al., 2000; Phillips et al., 2001) subunits, in agreement with the prevalence of the α4β2 subtype in the mammalian brain (Zoli et al., 2015). When expressed in *Xenopus laevis* oocytes or mammalian cell lines, mutant subunits tend to confer a gain-of-function phenotype, especially in the simulated heterozygote, because of increased receptor’s sensitivity to the agonist or other kinetic alterations (Becchetti et al., 2015). Several hypotheses concerning the nAChR-dependent pathogenetic mechanism have been proposed (Nobili et al., 2014). These are difficult to demonstrate considering that nAChRs are expressed in the brain at pre-, post-, and extra-synaptic locations (Dani and Bertrand, 2007), and they regulate both excitatory and inhibitory transmission (Becchetti et al., 2015). In prefrontal regions, heteromeric nAChRs exert a widespread stimulatory effect on glutamatergic transmission (Vidal and Changeux, 1993; Lambe et al., 2003; Aracri et al., 2013). These receptors also regulate GABAergic interneurons (Porter et al., 1999; Alkondon et al., 2000; Couey et al., 2007) although the expression of heteromeric nAChRs in these cells is more variable, depending on neuronal subtype and age (Porter et al., 1999; Couey et al., 2007; Aracri et al., 2010, 2017).

Understanding the nAChR-dependent pathogenesis of ADNFLE is made even more complex by the involvement of *CHRNA2*. Two mutations with opposite effects on the channel functioning were previously reported in the *CHRNA2* gene, coding for the nAChR α2 subunit. In particular, the p.Ile279Asn increases the receptor sensitivity to the agonists (Aridon et al., 2006), whereas the p.Ile297Phe mutation presents a strongly decreased current density as compared to the WT, but scarce alteration of the conductive properties and the sensitivity to nicotine (Conti et al., 2015). Mutations in the *CHRNA2* are rare in the Italian ADNFLE population (Combi et al., 2009). Hence, it is important to determine whether *CHRNA2* mutations can be a significant etiologic factor in sleep-related hypermotor epilepsy, and what is the prevalent pathogenetic mechanism. Here, we report the third *CHRNA2* mutation detected in an ADNFLE patient, showing a loss of function effect when expressed in human cell lines.

## Materials and Methods

### Sample Composition and Genetic Analysis

The de-identified DNA of three individuals (one affected by NFLE and his parents) was isolated from leftover venous blood samples. Clinical samples and data were collected according to Italian authority laws on privacy protection (G.U. n. 72 26/03/2012) and genetic data (G.U. n. 159 11/07/2011), in compliance with the General Data Protection Regulation (EU Directive 2016/679) and with written consent from all subjects. The patient (>18 years old) and his parents signed a written informed consent form for the use of their biological materials for genetic and clinical research in accordance with the Helsinki declaration. No sensitive data are included in the manuscript.

A video-polysomnographic analysis allowed a correct diagnosis of NFLE.

Polymerase chain reactions (PCRs) were performed directly on 50–100 ng of genomic DNA in a 25 μL volume. Each reaction was performed using the PCR Master Mix (Promega, Madison, WI, United States). PCRs were carried out on Mastercycler Ep Gradient thermomodules (Eppendorf, Milan, Italy) under standard conditions. Primers used for amplification and sequencing reactions (Life Technologies, Inchinnan, Paisley, United Kingdom) were designed using the Oligo 6.0 software (Molecular Biology Insights Inc., Cascade, CO, United States) on the basis of the genomic sequences of known genes and can be provided upon request. Sequencing was carried out directly on both strands of purified PCR products by using the BigDye Terminator Cycle Sequencing kit v1.1 and an automated ABI-3130 DNA sequencer (Applied Biosystems, Foster City, CA, United States). ChromasPro v1.34 (Technelysium Pty Ltd.) software was used for mutation detection. The pathogenicity was predicted using PolyPhen-2^1^, SIFT^2^, and MutationTaster^3^ bioinformatic tools.

### Plasmid Constructs and Expression Vectors

Four F2A system-based tricistronic vectors for the expression of either the α2/β2 or the α2/β4 receptors, both in the presence or absence of the *CHRNA2* mutation were obtained following a strategy similar to those previously reported by Ryan and Drew (1994). To facilitate detection of the transfected cells, each vector also encoded for the e-GFP (enhanced green fluorescent protein) as a valuable reporter molecule. Briefly, the e-GFP coding sequence (CDS) was amplified without the stop codon and cloned into a *Bam*HI/*Bgl*II-digested pCX plasmid, to produce the pCX-eGFP (deltaTAG) vector. The first F2A sequence (F2A1), obtained as previously described (De Giorgi et al., 2015), was ligated by directional cloning downstream the eGFP sequence into the pCX-eGFP (deltaTAG) plasmid. The *CHRNA2* CDS (NCBI: NM_000742.3) was PCR-amplified removing the stop codon and cloned downstream the F2A1 sequence. The second F2A sequence (F2A2) was first amplified and then ligated in frame downstream the *CHRNA2* sequence in order to obtain the pCX-eGFP-F2A1-CHRNA2(WT)-F2A2 plasmid. Finally, the CDS of either *CHRNB2* (NCBI: NM_000748.2) or *CHRNB4* (NCBI: NM_000750.4) sequences were PCR-amplified including the stop codon and cloned into the *Afl*II-linearized pCX-eGFP-F2A1-CHRNA2(WT)-F2A2 plasmid acceptor downstream the F2A2 sequence generating the final constructs, named pCX-eGFP-F2A1-CHRNA2(WT)-F2A2-CHRNB2 and pCX-eGFP-F2A1-CHRNA2(WT)-F2A2-CHRNB4, respectively. For each PCR amplification, specific restriction sites were added at the 5′-end of both sequences to allow the directional cloning and each PCR product was firstly cloned into a pGEM T-Easy vector (Promega) as intermediate plasmid.

The p.Tyr252His (c.754T>C) *CHRNA2* mutation was introduced by Quick Change II XL Site Directed Mutagenesis Kit (Stratagene, La Jolla, CA, United States) into both pCX-eGFP-F2A1-CHRNA2(WT)-F2A2-CHRNB2 and pCX-eGFP-F2A1-CHRNA2(WT)-F2A2-CHRNB4 constructs, in order to obtain pCX-eGFP-F2A1-CHRNA2(MUT)-F2A2-CHRNB2 and pCX-eGFP-F2A1-CHRNA2(MUT)-F2A2-CHRNB4 plasmids, respectively. All the intermediate and final constructs were verified by sequencing analyses performed on both strands using an automated ABI-3130 DNA sequencer (Applied Biosystems, Foster City, CA, United States). All plasmids were purified using the QIAGEN Plasmid Maxiprep kit (QIAGEN, Hilden, Germany) following the suggested protocol and resuspended in water.

### Culture and Transfection Procedure

Plasmids expressing wild-type (WT) or mutant α2β2 or α2β4 were transiently transfected in HEK293 cells (TsA subclone; American Type Culture Collection) as reported (Conti et al., 2015). In brief, cells were cultured in DMEM high glucose (Dulbecco’s modified Eagle medium high glucose; HyClone Laboratories, Logan, UT, United States) supplemented with 10% fetal calf serum (HyClone) and 2 mM L-glutamine, at 37°C and 5% CO_2_. For patch-clamp experiments, cells were seeded onto 35-mm culture dishes. Transfection was carried out with Lipofectamine 2000 (Life Technologies). To simulate the heterozygous state, equal amounts of WT and mutant plasmids were cotransfected. The DNA concentration in the transfection mixture was 1.33 ng/μL. Cells were incubated with the transfection mixture for 5 h, at 37°C, and kept at 30°C in 5% CO_2_ during the 24 h preceding the electrophysiological recordings, to enhance the surface receptor density (Cooper et al., 1999).

### Patch-Clamp Recording

Chemicals and drugs for intra- and extracellular solutions were purchased from Sigma-Aldrich. The extracellular solution contained (mM): NaCl 130, KCl 5, CaCl_2_ 2, MgCl_2_ 2, HEPES 10, and D-glucose 5 (pH 7.3). Patch pipettes contained (mM): K-gluconate 140, KCl 5, MgCl_2_ 1, BAPTA-KOH 0.5, HEPES 10, NaGTP 0.3, and MgATP 2 (pH 7.3). Stock solutions of nicotine (10 mM) were prepared weekly in our extracellular solution and kept refrigerated; acetylcholine (10 mM) and atropine (1 mM) were dissolved in extracellular solution, aliquoted and frozen until usage. Extracellular solutions with the appropriate agonist concentration were prepared daily; pH was always checked after nicotine addition.

Whole-cell currents were registered 36–72 h after transfection, with an Axopatch 200B amplifier (Molecular Devices, Sunnyvale, CA, United States), at room temperature. Micropipettes (3–5 MΩ) were pulled from borosilicate capillaries (Corning Inc., NY, United States) with a P-97 Flaming/Brown Puller (Sutter Instruments, Novato, CA, United States). Cell capacitance and series resistance (up to 75%) were always compensated. When necessary, the cell capacitance value thus measured was used to calculate the cell current density (i.e., the peak whole-cell current at a given V_m_ and agonist concentration was divided by the cell capacitance). Because the cell capacitance is proportional to the cell surface area, the calculated values are proportional to the current per unit area. Fluorescent cells were identified with an inverted Eclipse TE200 microscope (Nikon) equipped with a TE-FM epifluorescence attachment. Currents were low-pass-filtered at 2 kHz and acquired online at 10–20 kHz with pClamp nine hardware and software (Molecular Devices). Drugs were applied with an RSC-160 Rapid Solution Changer (Bio-Logic Science Instruments, Claix, France).

Patch-clamp data were analyzed with OriginPro 9 (OriginLab), as previously described (Brusco et al., 2015). Theoretical curves best fitting the data were calculated by a Levenberg-Marquardt algorithm. The concentration-response data were fitted by using a two-components Hill-type equation (Covernton and Connolly, 2000), as follows:

$$\frac{I_{L}}{I_{max}}\;=\;\;\frac{A}{1+{\left(\frac{EC_{50high}}{\left[L\right]}\right)}^{nH1}}\;\;+\;\;\;\;\;\;\frac{1-A}{1+{\left(\frac{EC_{50low}}{\left[L\right]}\right)}^{nH2}}\;\;\;\;\;\;\;\;\;\;\;\;\;\;\;\;\;\;\;\;\;\;\;\;\;\;(1)$$

where I_max_ is the maximal current, I_L_ is the current at a given concentration L of agonist, A is the fraction of receptors in the high-affinity state; EC_50high_ and EC_50low_ are the agonist concentrations producing the half-maximal effect for the high and low affinity components, respectively; nH1 and nH2 are the Hill coefficients for the two components.

### cDNA Synthesis and Real-Time Quantitative PCR

Total RNA was isolated from cultured cells using Direct-zol RNA MiniPrep (Zymo Research) and eluted in water. One microgram of the total extracted amount of RNA was subsequently treated with DNase I and reverse-transcribed using SuperScript VILO cDNA Synthesis Kit (Invitrogen). The first-strand cDNA was used as a template for real-time PCR (RT-PCR) using a human *CHRNA2* specific primer pair (Fw 5′-GCTAAAACAGGAGTGGAGCG-3′ and Rv 5′-TCGAAGGGGAAGAAGGTGAC-3′) and EvaGreen fluorescent dye (Bio-Rad). PCR reaction was performed using a CFX96 Real-time system (Bio-Rad) sequence detector. Data, normalized to eGFP transcript levels, are expressed as fold change value respect to the untransfected cells according to the 2^-[ΔΔC(q)]^ algorithm.

### Western Blotting

The anti-α2 and β4 Abs were produced in rabbits immunized with the human peptides CHPLRLKLSPSYHWLESNVDAEEREV (α2) and GPDSSPARAFPPSKSCVTKPEATATSPP (β4), respectively, affinity purified and characterized as previously described (Mazzo et al., 2013).

SDS-PAGE and blotting were carried out by standard procedures. In brief, 20 μg of proteins obtained from HEK 293 cells transfected with α2β4, α2^Tyr252His^β4, or from non-transfected HEK293 cells were loaded separated by means of SDS-polyacrylamide gel electrophoresis using 9% acrylamide, and electrophoretically transferred to nitrocellulose membranes with 0.45 mm pores (Schleicher and Schull, Dassel, Germany). The blots were blocked overnight in 4% non-fat milk in Tris-buffered saline, washed in a buffer containing 4% non-fat milk and 0.3% Tween 20 in Tris-buffered saline, and incubated for 2 h with the primary antibody at the concentration of 5 μg/ml. They were then incubated for 1 h with the appropriate secondary antibody (anti-rabbit Ly-Cor IRDye800RD). After washing, the membranes were dried overnight in the dark at room temperature. The IR signal was measured using an Odyssey CLx – Infrared Imaging System. The signal intensity of the Western blot bands was quantified using iStudio software.

### Radioligand Binding Assays

(±)-[^3^H]Epibatidine (specific activity of 56–60 Ci/mmol) was purchased from Perkin Elmer (Boston, MA, United States). Non-radioactive epibatidine was purchased from Sigma-Aldrich. Saturation experiments were performed by incubating aliquots of membranes from HEK293 cells expressing α2β4 or α2^Tyr252His^β4 nAChR with 0.01–5 nM concentrations of (±)-[^3^H]Epibatidine (Perkin Elmer) overnight at 4°C. Non-specific binding was determined in parallel by incubation in the presence of 100 nM unlabeled epibatidine. After incubation, the samples were filtered on GFC filters soaked in 0.5% polyethyleneimine and washed with 15 mL ice-cold phosphate buffered saline (PBS) and the filters were counted for radioactivity in a β counter.

### Statistical Analysis

Data are generally given as mean values ± standard error of the mean, with n representing the number of experiments (tested cells, in the case of patch-clamp experiments). Statistical comparisons between two populations of data were carried out with a Student’s *t*-test for unpaired samples, after checking for data normality (Kolmogorov–Smirnov test) and variance homogeneity (*F*-test). The Welch correction was applied in case of non-homogeneous variances. Multiple comparisons were carried out with one-way ANOVA, followed by Tukey *post hoc* test, after checking for data normality (Kolmogorov–Smirnov test) and variance homogeneity (Brown–Forsythe test). The level of statistical significance was set at *p* < 0.05. Data from saturation binding assays were evaluated by saturation binding curve-fitting procedures using GraphPad Prism version 6 (GraphPad Software, Inc., CA, United States).

## Results

### Clinical and Neurophysiological Studies in the Proband Carrying the p.Tyr252His *CHRNA2* Mutation

A 19-year-old right-handed man was referred for nocturnal episodes with abnormal motor-behavioral phenomena occurring several times every night. The episodes started at the age of 13 years. The majority of episodes were characterized by sudden vocalization with grunting followed by dystonic posturing; sometimes (2–3 episodes for week) a deambulatory behavior was reported. There was a family history of nocturnal confusional arousals in the mother during her adolescence: confusional arousal episodes occurred in the first part of the night (1–5 episodes for week, from age 13 to 16 years), in these episodes (5–20 s in duration) the mother sat up in bed and looked around in a confused manner.

Scalp EEG monitoring during wakefulness as well as the contrast-enhanced brain magnetic resonance imaging (MRI) were normal. Neurological examination was also normal.

**Figure 1:**
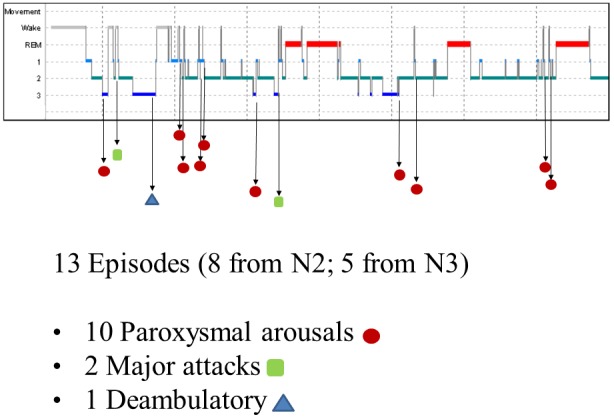
Hypnogram of the patient with the distribution of nocturnal episodes.

The nocturnal video-PSG recording showed 13 episodes, 8 in stage N2 and 5 in stage N3. Ten of these were classified as paroxysmal arousals, characterized by sudden arousals (5–8 s in duration) with stereotyped movements of arms and vocalization. Two episodes (16 and 19 s in duration, respectively) characterized by asymmetric dystonic posturing were classified as major attacks. The last episode was a deambulatory behavior with frightened expression and fear. Figure 1 shows the hypnogram with the distribution of nocturnal attacks registered in one night.

The EEG before, during and after the episodes did not show any epileptiform activity, but in eight episodes showed ictal rhythmic slow activity over anterior areas. A marked reduction of the nocturnal episodes was observed with the administration of carbamazepine (600 mg/day, single bedtime dose).

### Mutation Screening

**Figure 2:**
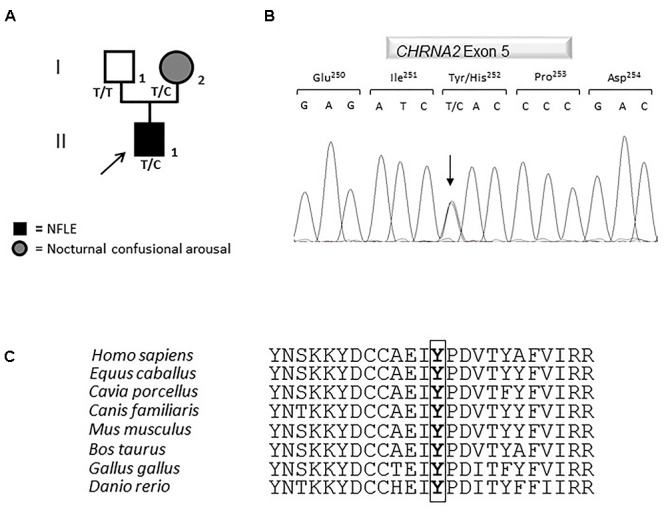
Genetics. **(A)** Pedigree of the family in which the mutation has been identified. The arrow points to the proband. Genotypes are shown. T/T: wild-type (WT) genotype; T/C: heterozygous genotype. Squares indicate males while circles indicate females. The legend of each kind of symbol filling is reported. **(B)** Electropherogram from the proband heterozygous for the transition c.754T>C (RefSeq NM_000742.3) that corresponds to the missense mutation p.Tyr252His. **(C)** Amino acid multiple alignment of the α2 subunit of the nAChR sequence displaying evolutionary conservation of Tyrosine Y residue across species.

The coding region, intron-exon boundaries and UTRs of *CHRNA4*, *CHRNB2*, *CHRNA2*, *CRH*, *KCNT1* genes previously associated with ADNFLE were amplified and Sanger sequenced. This work revealed that the proband is a heterozygote for a missense mutation in the *CHRNA2* gene (Figure 2A). Nucleotide numbering from here onward is according to cDNA position (GenBank accession number NM_000742.3 starting from the first nucleotide of the ATG start codon).

The mutation consists of a T>C transition at cDNA position 754 (c.754T>C), which leads to a non-conservative Tyr to His change at position 252 (p.Tyr252His, according to the Human Genome Variation guidelines) in the α2 subunit of the nAChR. Electropherograms of exon five encompassing the mutation are shown in Figure 2B. The variation was not reported yet and it was located in the N-terminal domain, in a highly conserved region (Figure 2C) involved in the acetylcholine binding.

A segregation analysis was performed and the mutation was found in the heterozygous state also in the affected proband’s mother, while it was absent in the healthy father (Figure 2A). The mother reported to have been affected by nocturnal confusional arousal in her adolescence but no clinical examinations are available to evaluate the existence of an undiagnosed NFLE phenotype.

Since the mutation had never been studied from a functional point of view, we performed a bioinformatic analysis using Polyphen-2, SIFT or MutationTaster, in order to predict its possible effect on the channel functionality. The p.Tyr252His was predicted to be probably damaging by all these tools. This would be related to the fact that the mutation causes the substitution in an important functional domain of a polar but not charged amino acid with an aromatic R group (the Tyr) with another (the His) with a positively charged R group. Because the mutation was not reported yet, we decided to study its effects on the channel properties.

### The Mutation Did Not Alter the Transcription Level of the Gene in HEK293 Cells

**Figure 3:**
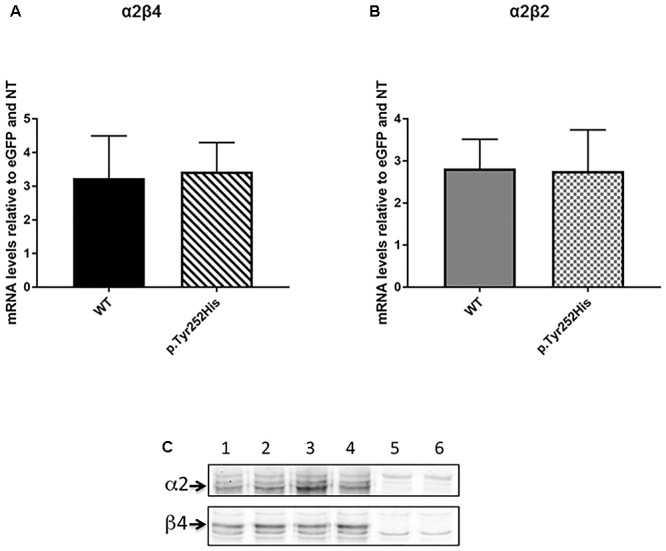
*CHRNA2* mRNA levels detected by real-time quantitative PCR in HEK293 cells transfected with tricistronic vectors containing either the wild-type (WT) or the mutant *CHRNA2* (p.Tyr252His) in combination with *CHRNB4* **(A)** or *CHRNB2* **(B)** cDNAs and the eGFP reporter. Data represent the mean ± SEM (*n* = 3) and are expressed as fold increase of mRNA levels normalized to eGFP transcript levels and to non-transfected HEK293 cells (NT). **(C)** Equal amount of membranes proteins from HEK293 cells transfected with either α2β4 (lanes 1 and 2), or α2^Tyr252His^β4 (α2^∗^β4; lanes 3 and 4) or untransfected (lanes 5 and 6) were separated on 9% acrylamide SDS gels, electrotransferred to nitrocellulose, probed with 5 μg/ml of the anti-α2 or anti-β4 primary Ab (as indicated), and then incubated with the secondary Ab (anti-rabbit Ly-Cor IRDye800RD, dilution 1:20000). The IR signal was measured using an Odyssey CLx – Infrared Imaging System and the signal intensity of the WB bands of the α2 and β4 subunits was quantified using iStudio software. The arrows indicate the α2 or β4 subunits.

In order to evaluate the possible effects of the newly identified p.Tyr252His mutation in the *CHRNA2* gene, we engineered an F2A-based multicistronic plasmid encoding for the different subunits of nAChR and a reporter gene for transfecting HEK293 cells. Firstly, we tested if the mutation could affect affect the transcription of the α2 subunit. To this extent, the correct transcription of the expression vectors in HEK293 cells was verified by RT-PCR. No differences in transcription levels were observed between the WT and mutant *CHRNA2* using both tricistronic vectors. In particular, the mRNA levels were 3.243 ± 1.249 (WT *CHRNA2*; *n* = 3) vs. 3.433 ± 0.864 (mutant *CHRNA2*; *n* = 3; *p* > 0.05, with unpaired *t*-test), in combination with *CHRNB4*. The corresponding values for the combination with *CHRNB2* were 2.860 ± 0.696 (WT *CHRNA2*; *n* = 3) vs. 2.760 ± 0.979 (mutant *CHRNA2*; *n* = 3, *p* > 0.05 with unpaired *t*-test). These results are shown in Figure 3A,B, and indicate that p.Tyr252His *CHRNA2* did not affect nAChR gene transcription and the plasmids gave similar levels of expression in our cells.

### α2^Tyr252His^ Did Not Alter Membrane Expression of α2β4

In order to determine whether the mutation could affect different level of expression of receptor subtypes, we then performed Western Blotting (WB) analysis by loading on the gel the same amount of membrane proteins. Figure 3C shows the WB analysis of two separate samples of α2β4 (lanes 1 and 2), two samples of α2^Tyr252His^β4 (lanes 3 and 4) and two samples of untransfected HEK293 cells (lanes 5 and 6). The quantitative analysis of three independent preparations of WT α2β4 and α2^Tyr252His^β4 showed that the α2 and β4 subunit content was identical between cells transfected with α2β4 or α2^Tyr252His^β4 (Figure 3C).

### Patch-Clamp and Radioligand Assay Analysis

**Figure 4:**
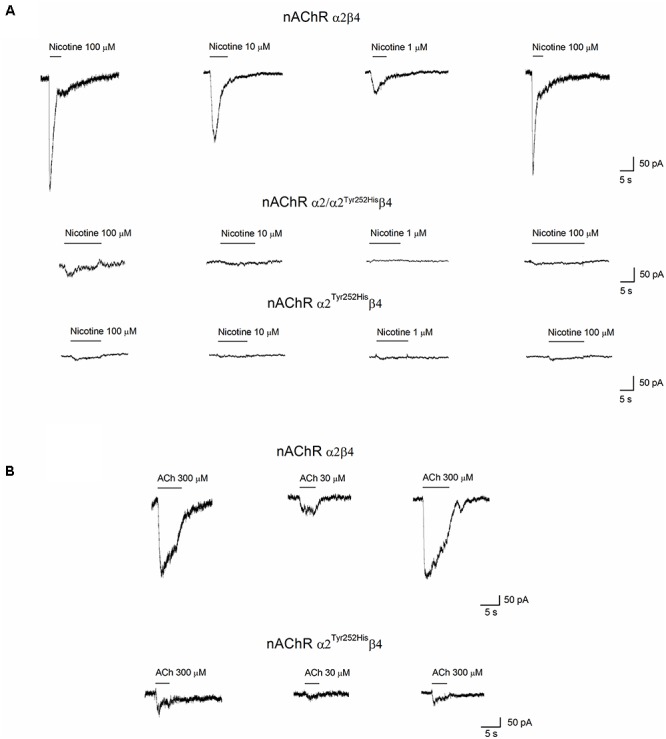
Whole-cell currents from nAChR receptors containing or not α2^Tyr252His^. **(A)** Representative whole-cell current traces elicited at –60 mV by the indicated concentration of nicotine, in cells expressing α2/β4 (wild type), α2^Tyr252His^/β4 (homozygote), or α2/ α2^Tyr252His^/β4 (heterozygote) receptors, as indicated. The bars above the current traces mark the time of nicotine application. The time gaps between consecutive traces represents about 2 min in the absence of agonist. **(B)** Same as **(A)**, except that ACh was used instead of nicotine, at the indicated concentrations. Cells expressed either α2/β4 (wild type), or α2^Tyr252His^/β4 (homozygote) receptors.

**Figure 5:**
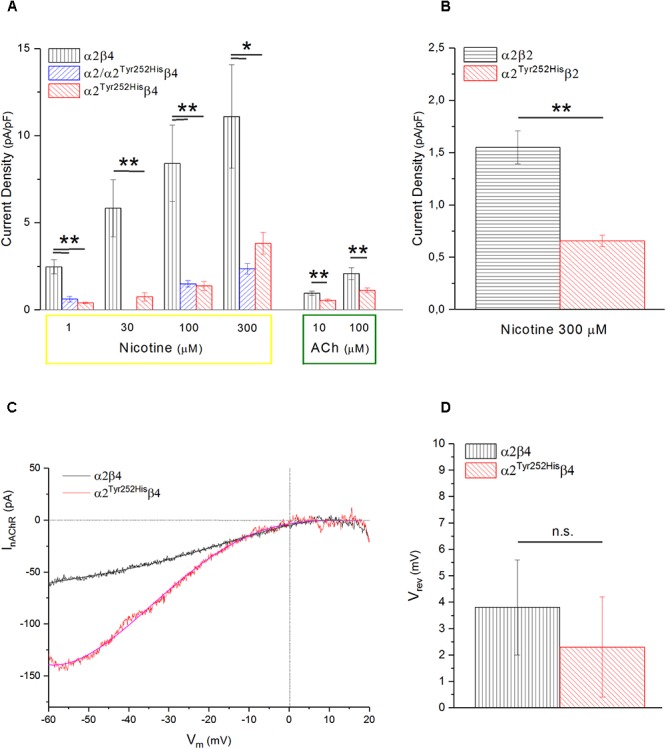
α2^Tyr252His^ decreases the maximal current density, without altering V_rev_. **(A)** Bars represent average peak whole-cell current densities measured at the indicated concentrations of nicotine or ACh, in cells expressing α2β4, α2^Tyr252His^β4, or α2^Tyr252His^/α2β4. The results of representative measurements are shown for 1 μM nicotine (*p* = 0.00005 between WT, *n* = 17, and homozygotes, *n* = 12; *p* = 0.0002 between WT and heterozygotes, *n* = 8), 30 μM nicotine (*p* = 0.0075; *n* = 9 for WT and *n* = 20 for homozygotes), 100 μM nicotine (*p* = 0.02 between WT, *n* = 9, and homozygotes, *n* = 22; *p* = 0.007 between WT and heterozygotes, *n* = 21), 300 μM nicotine (*p* = 0.02 between WT, *n* = 8, and homozygotes, *n* = 22; *p* = 0.01 between WT and heterozygotes, *n* = 21), 10 μM ACh (*p* = 0.0023; *n* = 17 for WT and 15 for homozygotes), 100 μM ACh (*p* = 0.010; *n* = 22 for WT and *n* = 19 for homozygotes), ^∗^*p* < 0.05; ^∗∗^*p* < 0.01. **(B)** Same as **(A)**, but for α2β2 and α2^Tyr252His^β4 receptors. For 1 μM nicotine (*p* = 0.0004; *n* = 10 for WT and *n* = 7 for homozygotes), 10 μM nicotine (*p* = 0.0008; *n* = 11 for WT and *n* = 7 for homozygotes), 300 μM nicotine (*p* = 0.0006; *n* = 12 for WT and *n* = 11 for homozygotes). **(B)** Same as **(A)**, but for α2β2 and α2^Tyr252His^β4 receptors, tested with 300 μM nicotine (*p* = 0.0006; *n* = 12 for WT and *n* = 11 for homozygotes), ^∗∗^*p* < 0.01. **(C)** Representative current traces for the indicated receptor type, obtained by stimulating the cell with 1 s voltage ramps (–60 to +20 mV), in the presence or absence of 600 μM nicotine. The background current was subtracted to the one obtained in the presence of nicotine. V_rev_ was estimated by fitting the currents with a polynomial function. **(D)** Average V_rev_ values measured in WT (*n* = 11) and mutant (*n* = 11) receptors. The reported values were not significantly different between WT and mutant (with unpaired *t*-test).

Whole-cell currents were elicited at -60 mV, by using nicotine or ACh. In Primate brain, the expression of α2 largely overlaps with that of both β2 and β4 (Han et al., 2000; Quik et al., 2000). Moreover, there is evidence of *in vivo* expression of α2β2^∗^ (Zoli et al., 2015), α2α4β2^∗^ (Quik et al., 2005), and α2β4^∗^ (Zoli et al., 1998). Therefore, we studied the functional effects of α2^Tyr252His^ on both α2β4 and α2β2 receptors. Representative current traces obtained from cells expressing α2β4 nAChRs are shown in Figure 4A (top panel). The maximal currents were repeatedly measured during the experiment, to check for possible activity rundown. Saturating nicotine concentrations (100–300 μM) elicited the typical inward current with desensitization. Consecutive agonist applications were spaced at least 2 min apart, to allow full channel recovery from desensitization. Lower agonist concentrations elicited smaller currents, with a slower desensitization. Similar experiments were carried out on cells expressing α2^Tyr252His^α2β4 receptors (simulated heterozygote; Figure 4A, middle panel), or α2^Tyr252His^β4 (homozygote; Figure 4A, bottom panel). The receptors containing α2^Tyr252His^ generally presented much lower current amplitudes, compared to the WT. Similar results were obtained by using the physiological agonist ACh, instead of nicotine. Representative current traces are shown in Figure 4B. When using ACh, atropine (1 μM) was added to the extracellular solution, to avoid the possible interference of muscarinic ACh receptors. To compare the current amplitudes obtained in cells with different surface areas, we report in Figure 5A the average peak whole-cell current densities (i.e., for each cell, the peak current was divided by the cell capacitance) obtained in the presence of the indicated concentrations of agonist, for the indicated α2β4 nAChR subtypes. The current density observed in the presence of nicotine was decreased by approximately 80% by α2^Tyr252His^, in both homozygous and heterozygous condition. In agreement with previous reports (Di Resta et al., 2010; Conti et al., 2015), the α2β2 nAChR subtype generally yielded lower functional expression in HEK293 cells, as compared to α2β4. Therefore, the maximal current densities for α2β2 receptors are reported for 300 μM nicotine (Figure 5B). In this case, the presence of α2^Tyr252His^ brought the peak current density from 1.55 ± 0.3 pA/pF (WT; *n* = 12), to 0.67 ± 0.1 pA/pF (homozygote; *n* = 11). Similar results were obtained with the physiological agonist ACh. The average current densities measured at 10 and 100 μM ACh for WT and mutant receptors are shown in Figure 5A. Full statistics are given in the figure legend.

To study whether α2^Tyr252His^ produced major alterations in the nAChR ion selectivity, we measured the reversal potential (V_rev_) of α2β4 and α2^Tyr252His^/β4 receptors, as previously described (Conti et al., 2015). In brief, current-voltage relations were obtained by applying 1 s voltage ramps between -60 and +20 mV, in the presence or absence of nicotine. Three ramps were usually averaged in either condition. Next, to isolate the nicotinic current, the background current obtained in the absence of nicotine was subtracted to the current recorded in the presence of nicotine. The resulting current-voltage relations were fit by polynomial functions, to estimate the nAChR V_rev_. In general, V_rev_ turned out to be close to 0 mV for both α2β4 and α2^Tyr252His^β4 receptors, in agreement with the typical V_rev_ observed in mammalian heteromeric nAChRs (Becchetti et al., 2015). Representative current traces and the average V_rev_ values estimated in a series of similar experiments are shown, respectively, in Figure 5C,D. These results suggest that major alterations in the ion selectivity are unlikely to be produced by α2^Tyr252His^.

**Figure 6:**
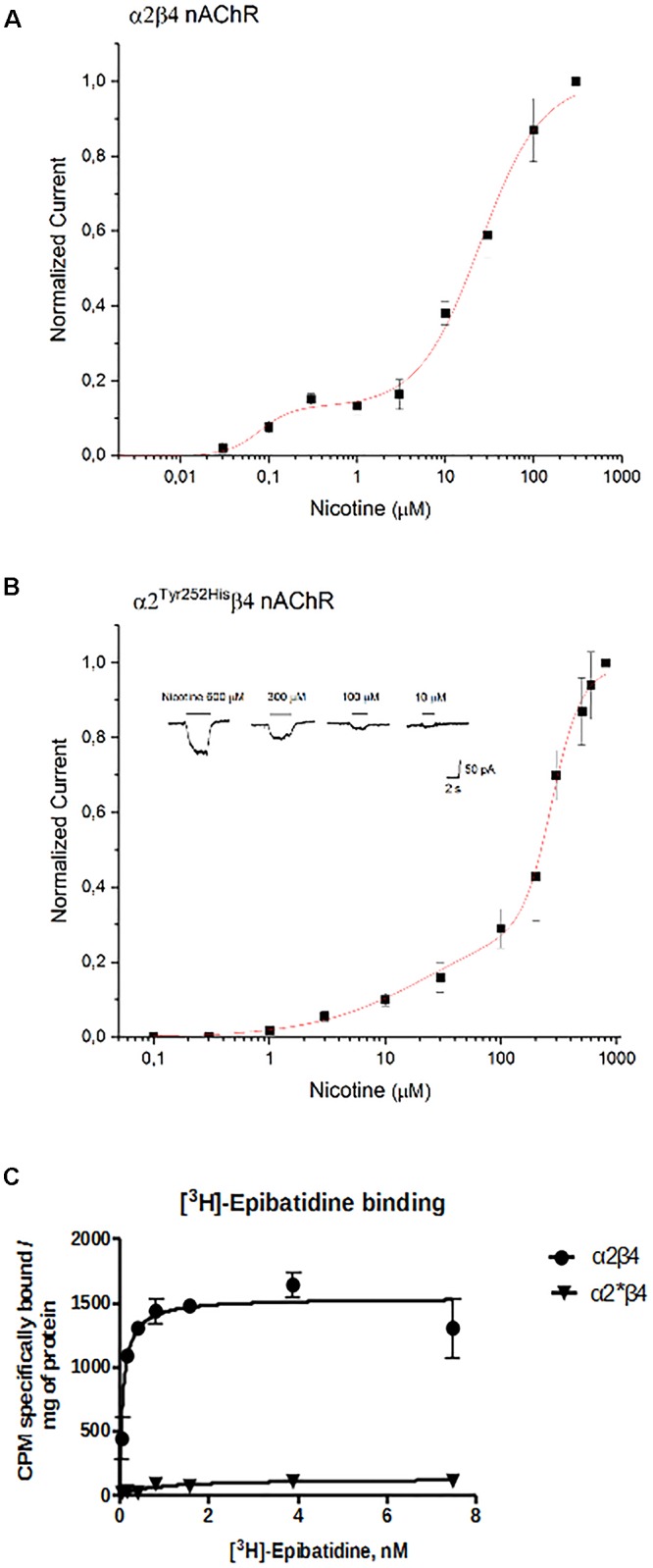
Concentration-response analysis. **(A)** Concentration-response relation derived from patch-clamp results for α2β4 receptors. Data points are average peak whole-cell currents, normalized to the current elicited by 300 μM nicotine in WT receptors. Continuous line is fit to equation (1). The relative estimated parameters were: EC_50high_: 0.08 ± 0.027 μM; EC_50low_: 24.7 ± 2.76 μM; nH1: 2.41 ± 2.6; nH2: 1.24 ± 0.15. **(B)** Same as **(A)**, for α2^Tyr252His^β4. In this case, peak currents are normalized to the current elicited by 800 μM nicotine. Representative currents are shown in the inset. Continuous line is fit to equation (1). The relative estimated parameters were: EC_50high_: 23.4 ± 23 μM; EC_50low_: 275.7 ± 12.5 μM; nH1: 0.87 ± 0.27; nH2: 3.44 ± 0.81. **(C)** Saturation binding experiments aimed to determine K_d_ and B_max_ of [^3^H]Epibatidine in cells transfected with α2β4, or α2^Tyr252His^β4 (α2^∗^β4), or non-transfected. Curves were obtained from two independent saturation experiments using a non-linear least squares analysis program using GraphPad Prism version 6.

The concentration-response curves for nicotine were obtained by applying different concentrations of agonist at -60 mV. The peak currents thus obtained were normalized to the current obtained at 300 μM (for α2β4), or 800 μM (for α2^Tyr252His^β4), and, respectively, plotted in Figure 6A,B. At higher agonist concentrations, the peak currents tended to decrease. This is also observed with other nAChR subtypes, and has been attributed to a blocked channel state at high concentrations of agonist (Maconochie and Knight, 1992). The presence of α2^Tyr252His^ strongly decreased the amplitude of the currents activated by nicotine, which were barely detectable at concentrations lower than 10 μM. In fact, α2^Tyr252His^ caused an approximately 10-fold right shift of the apparent EC_50_ of both the high and low affinity components of α2β4 receptors. In particular, EC_50high_ was ∼23 μM for α2^Tyr252His^β4, and ∼0.8 μM for α2β4 receptors, while EC_50low_ was ∼275 μM for α2^Tyr252His^β4, and ∼25 μM for α2β4 receptors. Full statistics are given in the figure legend. The patch-clamp results can be compared with the measurements carried out with [^3^H]Epibatidine (Figure 6C). The binding affinities (K_d_) of [^3^H]Epibatidine for transfected α2β4 and α2^Tyr252His^β4 subtypes, were determined by saturation binding experiments. The affinity (K_d_) of [^3^H]Epibatidine for the α2β4 or α2^Tyr252His^β4 nAChR subtypes were, respectively, 0.085 and 0.89 nM, and were derived from the average value of two independent [^3^H]Epibatidine binding saturation experiments.

In addition to the difference in K_d_, analysis of the saturation curves also showed that the B_max_ of [^3^H]Epibatidine binding (expressed as cpm specifically bound/mg of protein) is much lower for α2^Tyr252His^β4 receptors than for α2β4. In fact, fitting the saturation curves and calculating the cpm specifically bound by [^3^H]Epibatidine/mg of protein gave 1535 cpm for α2β4 and 127 for α2^Tyr252His^β4 (Figure 6C). Considering the WB results (Figure 3C), we conclude that the strong decrease produced by α2^Tyr252His^ on both B_max_ and maximal whole-cell currents can be attributed to a conspicuous decrease in the number of channels bound to the agonist.

## Discussion

In the present work, we reported a new *CHRNA2* mutation detected in an ADNFLE patient. When expressed in HEK293 cells, the receptors containing α2^Tyr252His^ displayed a marked reduction of whole-cell currents, as compared to WT receptors, in all experimental conditions. Such a decrease was paralled by a B_max_ decrease with [^3^H]-epibatidine. Moreover, the concentration-response curves determined by both methods showed that α2^Tyr252His^ produced an approximate 10-fold decrease in the apparent affinity for the tested agonists of the α2β4 subtype (Figure 6). The decrease in maximal current and B_max_ could be caused by a smaller single-channel conductance, a more negative V_rev_, a decrease of the average number of active channels onto the plasma membrane, or a combination thereof. Because V_rev_ was not altered by α2^Tyr252His^ and considering that Tyr252 is placed far from the pore region, we believe a major alteration of the channel’s conductive properties is unlikely. Moreover, neither subunits’ transcription nor membrane expression were altered by α2^Tyr252His^ (Figure 3). Therefore, we attribute the overall reduction in the maximal response to the agonist, accompanied by a right-shift of the activation curve, to a strong decrease of the affinity of the ligand binding site for the agonist. Based on subunit sequence and what is known about the 3D structure of human α4β2 nAChRs (Morales-Perez et al., 2016; Walsh et al., 2018), as well as the extracellular domain of human α2 subunits (Kouvatsos et al., 2016), Tyr252 results to be located in the pre-M1 functional loop C. A simple explanation of our results is that adding a positively charged histidine in the binding site would cause an electrostatic repulsion for the positively charged agonists, which would lead to a lower binding affinity. It is also possible that altering the local structure of the pre-M1 region could considerably increase the energy required to transduce the conformational change from the ligand binding site to the pore region. Fully discriminating between these (not mutually exclusive) possibilities would require extensive single channel data. Regardless of the mechanistic details, our results suggest that, in the case of α2^Tyr252His^, a dominant negative effect is probably responsible for the main pathophysiological consequences.

The functional features conferred to the nAChR by α2^Tyr252His^ resemble those previously observed with p.Ile297Phe (Conti et al., 2015), and differ from those of p.Ile279Asn (Aridon et al., 2006). These results support the notion that loss of receptor function may be a more common epileptogenic mechanism for mutant α2^∗^ nAChRs, as compared to other nicotinic subunits. We hypothesize that the reasons for this difference may depend on the different distribution of nAChR subunits in the brain. The specific role of each subunit is still uncertain (Zoli et al., 2015), and particularly so in the case of α2 (Baddick and Marks, 2011), despite its relatively widespread expression in the mammalian brain (Wada et al., 1989; Marks et al., 1992). Recent work in the mouse neocortex suggested that α2 nAChR subunits are specifically expressed in the Martinotti cells that project to layer I and can synchronize the thick-tufted pyramidal cells in layer V (Hilscher et al., 2017). The present uncertainties about the distribution of α2 subunits at the cellular level in the human brain prevent to bring the comparison too far. Nonetheless, we can hypothesize that a decreased cholinergic response in Martinotti cells could facilitate inhibition of these interneurons, which could lead to pyramidal cell excitation through rebound excitation (Becchetti et al., 2015; Hilscher et al., 2017).

## Author Contributions

CV, GC, CG, RG, AB, and RC conceived and designed the experiments. CV, GC, SM, CG, MM, and EC performed the experiments. CV, GC, SM, CG, MM, AB, and RC analyzed the data. LF-S, CG, AB, and RC contributed to reagents, materials, and analysis tools. CV, GC, CG, LF-S, AB, and RC wrote the manuscript.

## Conflict of Interest Statement

The authors declare that the research was conducted in the absence of any commercial or financial relationships that could be construed as a potential conflict of interest.

## Footnotes

1 http://genetics.bwh.harvard.edu/pph2/

2 http://sift.jcvi.org/

3 http://www.mutationtaster.org/
